# West Nile Virus Epidemic in Germany Triggered by Epizootic Emergence, 2019

**DOI:** 10.3390/v12040448

**Published:** 2020-04-15

**Authors:** Ute Ziegler, Pauline Dianne Santos, Martin H. Groschup, Carolin Hattendorf, Martin Eiden, Dirk Höper, Philip Eisermann, Markus Keller, Friederike Michel, Robert Klopfleisch, Kerstin Müller, Doreen Werner, Helge Kampen, Martin Beer, Christina Frank, Raskit Lachmann, Birke Andrea Tews, Claudia Wylezich, Monika Rinder, Lars Lachmann, Thomas Grünewald, Claudia A. Szentiks, Michael Sieg, Jonas Schmidt-Chanasit, Daniel Cadar, Renke Lühken

**Affiliations:** 1 Institute of Novel and Emerging Infectious Diseases, Friedrich-Loeffler-Institut, Federal Research Institute for Animal Health, 17493 Greifswald-Insel Riems, Germany; ute.ziegler@fli.de (U.Z.); martin.groschup@fli.de (M.H.G.); martin.eiden@fli.de (M.E.); markus.keller@fli.de (M.K.); friederike.michel@gmx.net (F.M.); 2 German Centre for Infection Research, Partner Site Hamburg-Luebeck-Borstel-Riems, 20359 Hamburg, Germany; 3 Institute of Diagnostic Virology, Friedrich-Loeffler-Institut, Federal Research Institute for Animal Health, 17493 Greifswald-Insel Riems, Germany; pauline.santos@fli.de (P.D.S.); dirk.hoeper@fli.de (D.H.); martin.beer@fli.de (M.B.); claudia.wylezich@fli.de (C.W.); 4 Bernhard Nocht Institute for Tropical Medicine, WHO Collaborating Centre for Arbovirus and Hemorrhagic Fever Reference and Research, 20359 Hamburg, Germany; carolin.hattendorf@bnitm.de (C.H.); philip.eisermann@gmail.com (P.E.); schmidt-chanasit@bnitm.de (J.S.-C.); danielcadar@gmail.com (D.C.); 5 Institute of Veterinary Pathology, Freie Universitat Berlin, 14163 Berlin, Germany; robert.klopfleisch@fu-berlin.de; 6 Small Animal Clinic, Department of Veterinary Medicine, Freie Universität Berlin, 14163 Berlin, Germany; kerstin.Mueller@fu-berlin.de; 7 Leibniz-Centre for Agricultural Landscape Research, 15374 Müncheberg, Germany; doreen.werner@zalf.de; 8 Institute of Infectiology, Friedrich-Loeffler-Institut, Federal Research Institute for Animal Health, 17493 Greifswald-Insel Riems, Germany; helge.kampen@fli.de (H.K.); birke.tews@fli.de (B.A.T.); 9 Department of Infectious Disease Epidemiology, Robert Koch Institute, 13353 Berlin, Germany; frankc@rki.de (C.F.); lachmannr@rki.de (R.L.); 10 Clinic for Birds, Small Mammals, Reptiles and Ornamental Fish, Centre for Clinical Veterinary Medicine, Ludwig Maximilians University Munich, 85764 Oberschleißheim, Germany; monika.rinder@vogelklinik.vetmed.uni-muenchen.de; 11 Nature and Biodiversity Conservation Union, 10117 Berlin, Germany; lars.lachmann@nabu.de; 12 Infectious Diseases and Tropical Medicine Clinic, Klinikum Chemnitz, 09116 Chemnitz, Germany; t.gruenewald@skc.de; 13 Leibniz-Institute for Zoo- and Wildlife Research (IZW), 10315 Berlin, Germany; szentiks@izw-berlin.de; 14 Institute of Virology, Faculty of Veterinary Medicine, Leipzig University, 04103 Leipzig, Germany; michael.sieg@vetmed.uni-leipzig.de; 15 Faculty of Mathematics, Informatics and Natural Sciences, Universität Hamburg, 20148 Hamburg, Germany

**Keywords:** West Nile virus, Germany, epizooty, epidemic, human, bird, horses, mosquitoes, transmission risk, zoonoses

## Abstract

One year after the first autochthonous transmission of West Nile virus (WNV) to birds and horses in Germany, an epizootic emergence of WNV was again observed in 2019. The number of infected birds and horses was considerably higher compared to 2018 (12 birds, two horses), resulting in the observation of the first WNV epidemy in Germany: 76 cases in birds, 36 in horses and five confirmed mosquito-borne, autochthonous human cases. We demonstrated that Germany experienced several WNV introduction events and that strains of a distinct group (Eastern German WNV clade), which was introduced to Germany as a single introduction event, dominated mosquito, birds, horse and human-related virus variants in 2018 and 2019. Virus strains in this clade are characterized by a specific-Lys2114Arg mutation, which might lead to an increase in viral fitness. Extraordinary high temperatures in 2018/2019 allowed a low extrinsic incubation period (EIP), which drove the epizootic emergence and, in the end, most likely triggered the 2019 epidemic. Spatiotemporal EIP values correlated with the geographical WNV incidence. This study highlights the risk of a further spread in Germany in the next years with additional human WNV infections. Thus, surveillance of birds is essential to provide an early epidemic warning and thus, initiate targeted control measures.

## 1. Introduction

West Nile virus (WNV, family *Flaviviridae*, genus *Flavivirus)* is maintained in a transmission cycle between birds as amplification hosts and mosquito vectors [1]. Spillover events have significant public health and veterinary relevance [2]. A total of 25% of the infected people develop West Nile fever (WNF) and become symptomatic (e.g., headache or muscle pain) [3]. Severe disease progressions manifesting as WNV neuroinvasive disease (WNND) are rare (<1%) [4]. These include syndromes of meningitis, encephalitis, and acute flaccid paralysis/poliomyelitis. Case-fatality rate of WNND is approximately 10% [5]. Age is the most important risk factor for WNND and a fatal disease outcome [2]. Thus, WNV circulation poses considerable risk for transfusion and organ transplantation safety [6].

WNV is distributed in wide areas of Europe. The main focus of WNV circulation is in south-eastern Europe and Italy [7]. However, low WNV activity is also observed in the neighboring countries of Germany (France, Austria, and Czech Republic). Therefore, over the last decade, different monitoring programs were implemented in Germany to screen for WNV RNA and antibodies in birds, horses, mosquitoes and chicken eggs [8,9,10,11,12]. In 2018, an epizootic emergence of WNV was observed in Germany for the first time [13]. All WNV-positive birds and horses were infected with the same WNV lineage 2 strain of the central European subclade II. WNV activity was detected in eastern Germany over a distance of almost 900 km (Munich to Rostock). At the same time, a large WNV outbreak was observed in south-eastern and southern Europe [7]. However, phylogenetic analysis in combination with the wide distribution in Germany indicates that WNV may have been introduced from the Czech Republic to Germany already before 2018 [13]. The emergence of WNV in Germany and the focus in the central part of eastern Germany was correlated with outstandingly high summer temperatures. As demonstrated for other European countries, WNV is probably predominantly transmitted by different native *Culex* species. *Culex pipiens* biotype *pipiens*, *Culex pipiens* biotype *molestus* and *Culex torrentium* from Germany were experimentally proven to be susceptible to WNV infection [14].

In this study, we report a WNV epidemic in Germany, 2019, triggered by an epizootic emergence among birds with spillover to horses and humans. Human and animal cases were located in the same area, showing a high WNV activity also in 2018. In both years, the region was characterized by suitable temperature conditions allowing a short extrinsic incubation period (EIP). Phylogenetic and phylogeographic analysis showed that Germany experienced several WNV introduction events. Several virus variants circulate in the affected German regions with Austria and Czech Republic as possible origins. The majority of the WNV strains involved in the German outbreak clustered together into a distinct and dominating group (Eastern German WNV clade) comprising of mosquito, bird, horse and human-related virus variants.

## 2. Materials and Methods 

### 2.1. WNV Screening of Birds, Horses and Mosquitoes

Since the first outbreak of Usutu virus (USUV) in Germany (2011/12), a nationwide bird surveillance network (living and dead birds) was set up to monitor for zoonotic arboviruses with a focus on WNV and USUV. In this context, a variety of dead birds and organ samples were submitted to the Bernhard Nocht Institute for Tropical Medicine and the national reference laboratory for WNV at the Friedrich-Loeffler-Institut (FLI) by the regional veterinary laboratories of the federal states of Germany, by the German Mosquito Control Association (KABS), the Nature and Biodiversity Conservation Union (NABU), citizens and independent bird clinics and zoological gardens. WNV infection in birds and horses is a notifiable disease in Germany if a recent infection is detected by a WNV-specific RT-qPCR result and/or a positive result of horses by IgM-ELISA, i.e., the detection of a fresh WNV infection. A previous vaccination of horses must be excluded. A positive IgG or neutralizing antibody detection is not notifiable in Germany.

Requests for the submission of dead birds were made via press releases of involved institutes and subsequent dissemination of the information by different kinds of media, including newspaper articles, television and radio. Total RNA from homogenized tissue samples (brain, liver, lung, or heart) was extracted and analyzed for the presence of flavivirus RNA by using a modified pan-flavivirus reverse transcription PCR [15] or WNV-specific reverse transcription quantitative PCR (RT-qPCR) [16]. Furthermore, all samples were also tested using the USUV-specific RT-qPCR described by Jöst et al. [17] (data not shown). 

Organ samples from affected horses were also tested by the RT-qPCR as stated above. In the case of diseased horses, often with neurological symptoms typical for WNV disease, the serum samples were screened by IgM-and/or IgG-ELISA (IDVet, Grabels, France) and positive samples were confirmed by differentiating virus neutralization tests to exclude cross-reacting flaviviruses (USUV, tick-borne encephalitis virus (TBE)) [12]. 

Following the first confirmed avian WNV case in the Tierpark Berlin (Wildlife Park) in 2019, mosquitoes were collected in that park by EVS (Heavy Duty Encephalitis Vector Survey) traps (BioQuip Products, Rancho Dominguez, CA, USA) equipped with dry ice as an attractant. Traps were continuously operated from mid-September to early October and emptied daily. Captured mosquitoes were morphologically identified to species or complex using the determination key by Becker et al. [18] and pooled with up to ten specimens per pool. Pools were homogenized and subjected to RNA extraction and WNV RT-qPCR as described above [16]. Positive samples were inoculated on C6/36 cells (L 1299, Collection of Cell Lines in Veterinary Medicine (CCLV), Friedrich-Loeffler-Institut, Greifswald – Insel Riems, Germany). Six days after inoculation, the supernatant of infected cultures was tested again with WNV RT-qPCR and the two samples with the lowest Ct-value were used for NGS analysis [19].

### 2.2. Risk of WNV Transmission 

The extrinsic incubation period (EIP) gives the time between ingestion of a pathogen via blood meals and the vectors’ ability to retransmit the pathogen. In contrast to other indices for transmission risk (e.g., field-measured infection rates of vectors), this approach is a theoretical risk assessment using information on the temperature-dependent EIP from the literature. However, EIP values give an approximation of virus transmission risk through the mosquito vector under local temperature conditions. Therefore, daily EIP values (EIP_d_) of WNV were calculated with the formula −0.132 + 0.0092 × temperature [13,20]. The day-to-day mean E-OBS temperature dataset v20.0e (July 2018 to August 2019) was downloaded from http://www.ecad.eu [21]. Data analysis and visualization was conducted with the program R [22] using the packages lubridate [23] and raster [24]. For the risk assessment, EIP_d_ values for the subsequent days were summed up until the virus development was completed (=EIP). For each grid cell and year, EIP values were averaged for the period from 15th July to 14th August (=EIP_ave_). 

### 2.3. Data Sets and Genome Characterization of WNV

**Figure 1:**
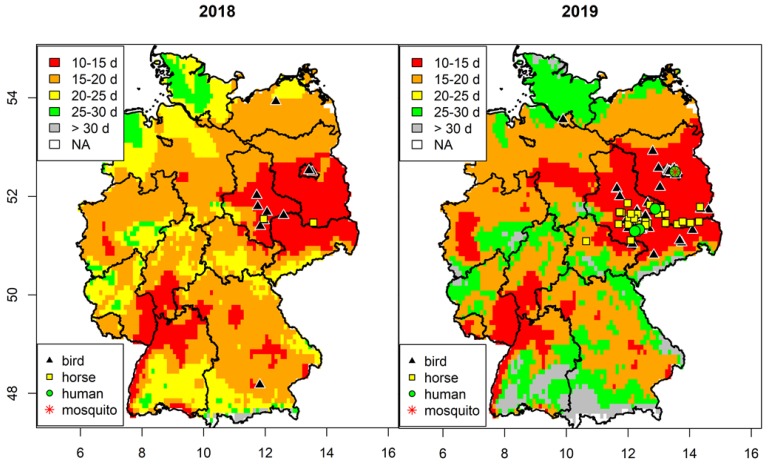
Spatial risk of West Nile virus (WNV) transmission in Germany. Average extrinsic incubation period between 15th July to 14th August 2018/2019 and distribution of WNV-positive birds, horses, humans and mosquitoes.

A total of 39 WNV genomes from birds, humans, mosquitoes and horses were newly acquired as part of this study (Table 1, Figure 1, Supplementary Table S1). The extracted viral RNA of WNV positive specimens was subjected to a next-generation sequencing (NGS) workflow [25], or to random RT-PCR amplification followed by library preparation by using the QIAseq FX DNA Library Kit (Qiagen, Hilden, Germany). They were normalized, sampled and sequenced using 150-cycle NextSeq550 Reagent Kits v2.5 (Illumina, San Diego, CA, USA) on a NextSeq550 platform (Illumina, San Diego, CA, USA) or the Ion Torrent S5 chemistry (ThermoFisher Scientific, Waltham, MA, USA) on an Ion Torrent S5 XL platform (ThermoFisher Scientific, Waltham, MA, USA). All whole genome sequences of WNV with known sampling time (year) and geographical origin (country) from Europe were retrieved from GenBank (n = 98) and combined with those sequenced in this study. Two data sets have been created: one containing all genomes from Europe incl. Germany, and a second one comprising the “Eastern German clade only.” Sequences were aligned using the MAFFT algorithm and then visually inspected in Geneious v2020.0.2 (https://www.geneious.com, Biomatters, Auckland, New Zealand). All sequences were confirmed as non-recombinant by the various methods for recombination detection implemented in RDP4 [26]. The obtained full-length recovered genome sequences of the WNV were submitted to GenBank or the European Nucleotide Archive (accession no. MN794935-MN794939, LR743421-LR743437, and LR743442-LR743458). 

### 2.4. Evolutionary Dynamics and Phylogeography of German WNV

Genomes obtained for the German WNV strains were compared with all European complete or near complete genomes sequences publicly available. For molecular clock phylogenetics, maximum clade credibility (MCC) trees were inferred using the Bayesian Markov chain Monte Carlo (MCMC) approach available in BEAST v1.10 [27]. Analyses were performed under the best fit nucleotide substitution model identified as GTR +Γ for the complete genome data set including “all European” genomes and TN93+Γ for the data set for “Germany only” using jModelTest 2 [28]. To search among maximum likelihood (ML) trees, we employed both nearest neighbor interchange (NNI) and subtree pruning and regrafting (SPR) branch swapping. To assess the robustness of each node, a bootstrap resampling process was performed (1000 replicates) again using the NNI branch-swapping method available in PhyML [29] (data not shown). We have employed the TempEst tool for an interactive regression approach to explore the association between genetic divergence through time and sampling dates [30]. In order to assess the spatiotemporal dynamics of WNV, the time to most recent common ancestor (tMRCA), evolutionary rate and the effective population dynamics of WNV was employed with a relaxed uncorrelated log normal and a strict molecular clock under a flexible demographic model (the coalescent Gaussian Markov Random field (GMRF) Bayesian Skyride) as the best demographic scenario detected. In all cases, each of the MCMC chain lengths was run for 5 × 10^7^ generations (with 10% burn-in) with subsampling every 10^4^ iterations to achieve convergence as assessed using Tracer v1.5 [31]. The MCC trees were visualized using FigTree v1.4.1 (http://tree.bio.ed.ac.uk/software/figtree/). To test the hypothesis that WNV was periodically introduced to Germany, a phylogeographic analysis was conducted using a discrete model attributing state characters represented by the detection locality of each strain and the Bayesian stochastic search variable (BSSV) algorithm implemented in BEAST v1.10 [27]. An MCC tree was summarized using TreeAnnotator v1.10. and visualized in FigTree v1.4.3. SpreadD3 v. 0.9.7.1 (https://rega.kuleuven.be/cev/ecv/software/SpreaD3_tutorial) was used to run BSSV analysis and generate Bayes factor (BF) and posterior probability (PP) to test for statistically significant epidemiological links between discrete sampling locations. The potential transmission networks within and between countries for NS5 WNV were inferred in PopART package v1.7.2 using median joining tree method with an epsilon of zero [32].

## 3. Results

### 3.1. Spatial Analysis of West Nile Virus Circulation

A total of 88 birds and 38 horses tested positive for WNV in 2018 (diagnosed between 28.8. and 9.10) and 2019 (diagnosed between 8.7. and 21.11) in Germany. In addition, five probably mosquito-borne human WNV cases were diagnosed with no history of travel to WNV-endemic countries within the last month. Except a single specimen (Hamburg, 2019), all WNV-positive animals originated from the eastern part of Germany with a distinct focus for the federal states Saxony-Anhalt, Saxony, Berlin and Brandenburg (Table 1, Figure 1). In addition, the targeted screening in the Tierpark Berlin revealed seven WNV positive *Culex pipiens* complex mosquito pools in 2019.

Low WNV activity was detected for the federal states Bavaria and Mecklenburg-Western Pomerania in 2018, which was not observed in 2019. WNV cases were found in Hamburg and Thuringia in 2019 for the first time. The number of positive birds and horses rose considerably in 2019 (76 birds and 36 horses) compared to 2018 (12 birds and two horses).

Especially in 2019, a large number of different bird species was affected (Table 2). A total of 52 birds (59.1% of all WNV-positive birds in 2018/2019) were held in captivity. From the total of 88 infected birds only four goshawks in private aviaries survived the infection. Of the 38 infected horses, 29 animals showed typical clinical symptoms, of which five horses died or were euthanized. Most of the other sick horses recovered in a very short time. Another nine horses were asymptomatic and were detected in the framework of additional investigations of holdings in relation to the clinical outbreaks. All 38 infected horses were positive in the IgM-ELISA and were therefore notified. 

The area with highest activity of WNV circulation was similar in 2018 and 2019, i.e., central-eastern Germany with most WNV-positive samples (mosquitoes, birds and horses) (Figure 1). In addition, all mosquito-borne, autochthonous human WNV cases were observed in this region. This matches the risk analysis based on the temperature conditions during summer, which indicates short EIP_ave_ (<15 days) for this area. The region along the Upper Rhine Valley (south-western Germany) was also characterized by low EIP_ave_ values, but no WNV circulation was detected in either year. Re-emergence of WNV was not observed for the most northern (Rostock) and southern (Poing) foci of WNV from 2018. This correlates with higher EIP_ave_ values for 2019 (>25 d; Poing: 28.4, Rostock: 26.2) compared to 2018 (<25 d; Poing: 21.6, Rostock: 19.6) for these areas, i.e., lower risk of WNV transmission.

### 3.2. Autochthonous Human WNV Cases

In September 2018, a 31-year-old male veterinarian developed flu-like symptoms after necropsy of a WNV-positive owl (https://promedmail.org/promed-post/?id=20181006.6074497). The laboratory confirmation was based on detection of an IgM response against WNV and a cross-reactive IgG response against WNV, which might be also the result of past vaccinations against TBE and yellow fever virus. The first mosquito-borne, autochthonous infection for Germany was confirmed in Leipzig, Federal State of Saxony on 20 September 2019. The 69-year-old male patient presented with WNND, received supportive treatment at the Infectious Diseases (ID) intensive care unit (ICU) between September 3rd and September 20th and was released with restitutio ad integrum. The laboratory confirmation was based on detection of WNV RNA in an early CSF, serum and urine sample and the detection of WNV IgM and IgG in serum samples. A second autochthonous case in Leipzig, an 81-year old male, was admitted to the ICU with presumptive diagnosis of pneumonia, then transferred to the ID-ICU and was found to have WNND confirmed by WNV IgM and IgG in serum samples as well as WNV RNA in CSF samples as early as from 19 September. He also recovered after 12 days of supportive care including mechanical ventilation without neurological sequelae. Both patients reported no history of travel to WNV-endemic countries and routes of non-vector borne transmission were excluded. While one patient had direct contact with the corpse of a Blue Tit (*Parus caeruleus*) five days before onset of fever, the other one reported no obvious contact with animals. Both patients had experienced multiple mosquito bites in the weeks before onset of illness. The third WNV infection was diagnosed on 24 September in a 46-year-old female patient from Berlin, Federal State of Berlin, who presented with West Nile Fever (WNF). The patient did not receive any treatment and recovered within two weeks. The laboratory confirmation was based on detection of WNV RNA in an early serum sample and seroconversion of WNV IgM and IgG in later serum samples. The fourth WNV infection was diagnosed retrospectively based on IgM and IgG detection in a serum sample on 16 October in a 44-year-old female patient from the district Wittenberg Federal State of Saxony-Anhalt. The patient was initially admitted to a local hospital with WNND-like symptoms on 10 September. After receiving supportive care, she was discharged on 17 September with restitutio ad integrum. The serological diagnosis was confirmed on 23 October by the detection of WNV RNA in a serum sample from the acute phase of infection. The fifth WNV infection was diagnosed on 23 October in a 24-year-old female patient from the district Leipzig, Federal State of Saxony, who presented with WNF (onset of symptoms 6 of October) and did not receive any treatment and recovered within one week. The laboratory confirmation was based on detection of WNV-specific IgM and IgG in a serum sample. As of 20 December, no further cases have been reported.

### 3.3. Genetic Characterization of German WNV

The genetic variations across the viral genome were low and homogenous (0.1%–0.7%) indicating that the analyzed WNV has maintained genetically stable since its first detection in 2018. The identity matrices for the genome and for individual genes were greater than 99.2%. The greatest variation was observed in the nonstructural genes coding for the NS1, NS2A, NS3 and NS5.

### 3.4. Phylogeny, Phylogeography and Spatiotemporal Dynamics of WNV

**Figure 2:**
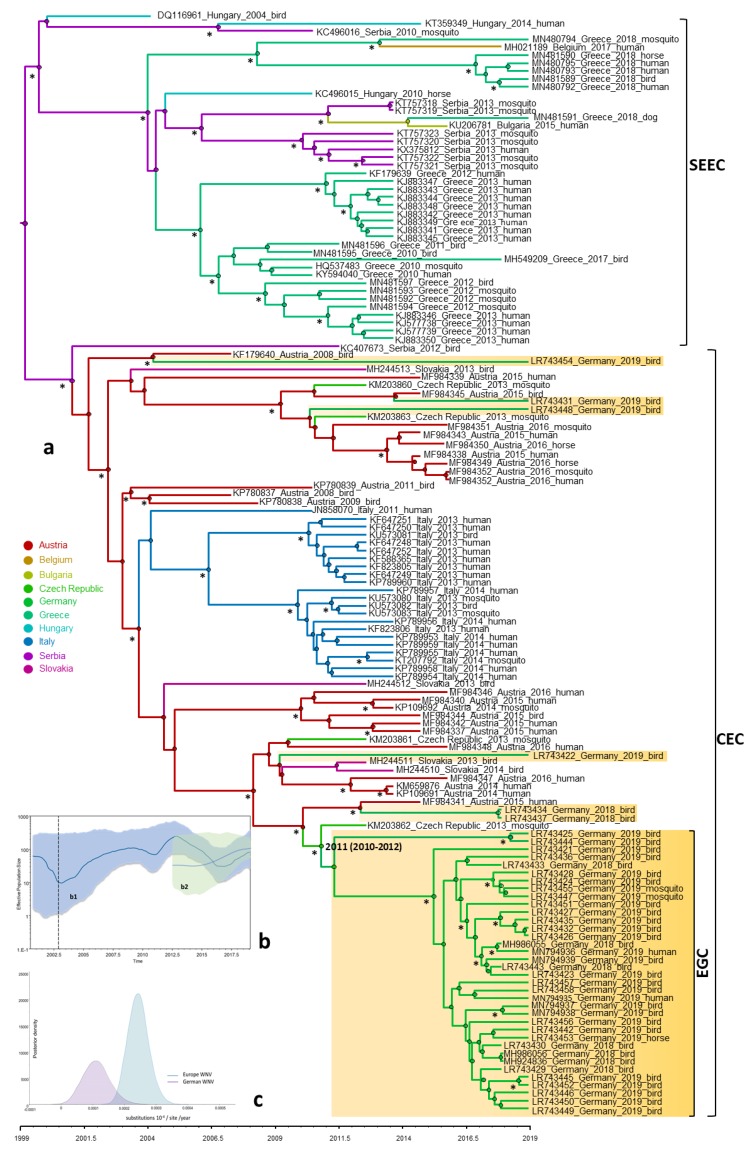
Bayesian maximum clade credibility (MCC) tree; (**a**) representing the time scale phylogeny; (**b**) effective population size; and (**c**) evolutionary rate of the European and German WNV lineage 2. The colored branches of MCC trees represent the most probable geographic location of their descendant nodes (see color codes); (**a**) the main clades are indicated to the right of the tree (SEEC, South Eastern European clade; CEC, Central and Eastern European clade), including the newly proposed German clade (EGC, Eastern German clade). Time is reported in the axis below the tree and represents the year before the last sampling time (2019). The German WNV strains sequenced in this study are highlighted. The estimated tMRCA of German WNV strains of EGC clade is shown with 95% posterior time intervals in parentheses. Bayesian posterior probabilities (≥90%) and 1000 parallel maximum likelihood bootstrap replicates (≥70%) are indicated at the nodes (asterisks); (**b**) temporal variation in the effective population size of the European WNV lineage 2; (**b1**) and EGC; (**b2**) estimated using the coalescent Gaussian Markov Random field (GMRF) Bayesian Skyride model of polyprotein sequences. The Bayesian Skyride plot represents temporal variation in the virus effective population size (Ne) through time. The blue line represents the median Ne estimate and the shaded area corresponds to the 95% high-probability density (HDP) intervals; (**c**) evolutionary rate estimates with 95% credible intervals for the distribution of evolutionary rates observed for the whole European WNV lineage 2 and for WNV from the 2018–2019 German epidemic.

**Figure 3:**
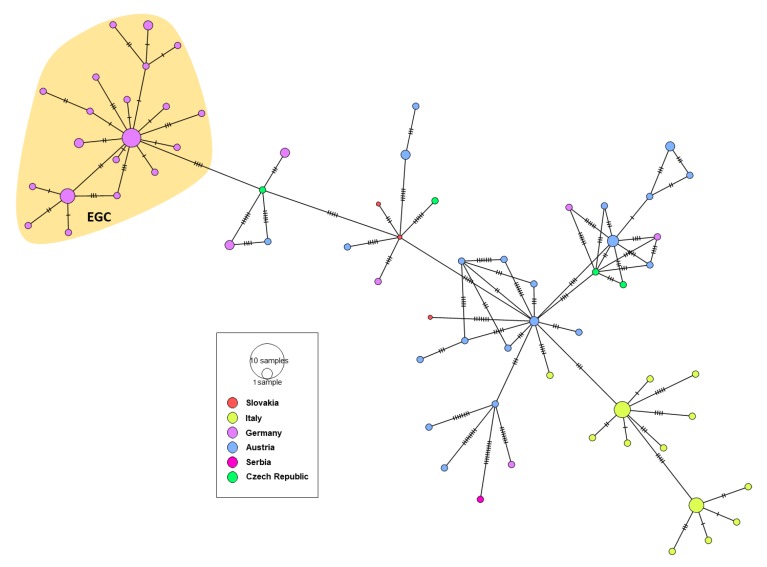
A median-joining haplotype network constructed from complete WNV NS5 gene alignment of the Central European WNV clade (CEC). Each colored vertex represents a sampled viral haplotype, with different colors indicating the different country of origin. The size of each vertex is relative to the number of sampled viral strains and the dashes on branches show the number of mutations between nodes. The Eastern German clade (EGC) is highlighted.

In order to investigate the evolutionary relationship and origin of WNV in Germany, a Bayesian MCMC sampling method and ML method were implemented. Similar topologies inferred by ML (not shown) and Bayesian MCC phylogenies of the European WNV lineage 2 data set revealed that all European strains fell into two distinct highly supported groups designated as Southeastern European clade (SEEC) and Central and Eastern European clade (CEC). All WNV strains from Germany fell into the CEC (Figure 2). The detailed analysis of the CEC showed that the German strains clustered in six distinct subclades (Figure 2) of which four consisted of singleton strains (WNV strains ED-I-155_19/ LR743422, ED-I-177_19/ LR743431, ED-I-201_19/ LR743448 and ED-I-205_19/ LR743454) associated with Austrian relatives (Figure 2). However, the majority of the WNV strains from Germany clustered into a well defined monophyletic group designated as Eastern German clade (EGC). The EGC is also notable for a star-like structure in which several subclades connect viruses sampled from multiple locations and time points (Figure 2a and Figure 3). These and other findings revealed that the genetic diversity of WNV in Europe is shaped primarily by in situ evolution rather than by extensive migration. No specific phylogenetic clustering and differences between the WNV strains from birds, horses, mosquitoes and humans in Germany were observed. The genetic variations of WNV combined with sample collection dates and locations can help to identify the possible source and the evolutionary history of the newly emerging viral variants. In order to assess viral migration and explore the origin of the WNV outbreaks in Germany, a discrete-trait phylogeography analysis was used to reconstruct the WNV movements between European countries and within Germany. Both data sets (European and German strains only, EGC) exhibited strong temporal signals (R^2^ = 0.31 for the “European” data set and 0.19 for the “German strains only,” *p* < 0.001). The coefficient of rate variation supports the use of a strict clock model for European data set and a relaxed clock for the data set “German strains only” (data not shown). The Bayesian MCC tree showed that although the WNV diversity in the “German strains only” group appears to have emerged in the last four years, the phylogeny of CEC which includes EGC suggests relative long-term circulation and evolution in Central Europe (Figure 2).

In further detail, the phylogeographic analysis suggests at least six distinct introductions of WNV into Germany from neighboring countries. It is predicted that all viral clade evolution events occurred during the last 16 years (Figure 2a). It should be noted that unlike its designation may suggest, the EGC can have developed in the wider southeastern and central European hemisphere and may have been translocated only later to Eastern Germany. Sequencing a larger number of more current WNV strains from e.g., Austria, the Czech Republic, and Poland would help to answer the circumstances of when and what in regard to the development of the East German Clade variants. Overall, the number of recent whole genome sequences is limited and should be markedly increased using NGS-based approaches.

**Figure 4:**
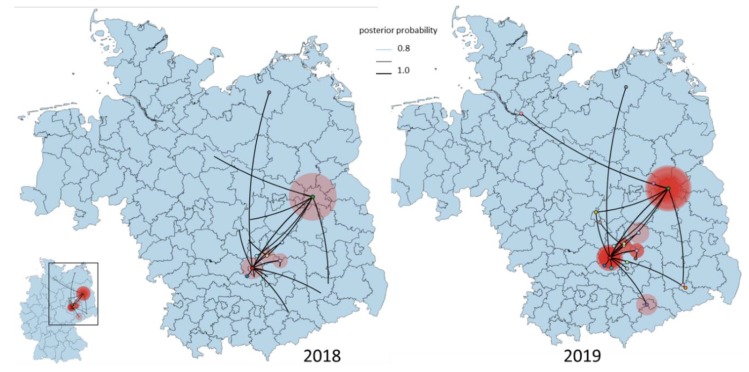
Temporally framed snapshots of the dispersal patterns (2018–2019) among regions in Germany for the Eastern German WNV clade. Lines between locations represent branches in the Bayesian maximum clade credibility (MCC) tree along which the relevant location transition occurs. Circle diameters are proportional to the square root of the number of MCC branches maintaining a particular location state at each time point.

**Figure 5:**
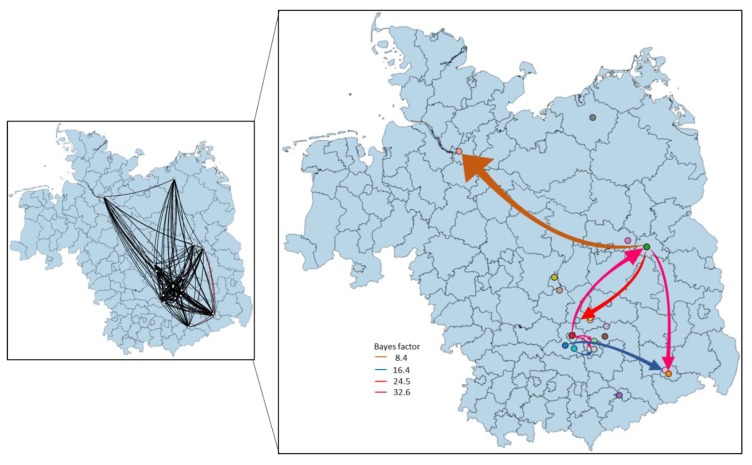
Calculated migration pattern of WNV between German locations based on Bayes factor test for significant non-zero rates. The arrows indicate the origin and the direction of migration between locations, while the colors indicate the strength of the connections.

**Figure 6:**
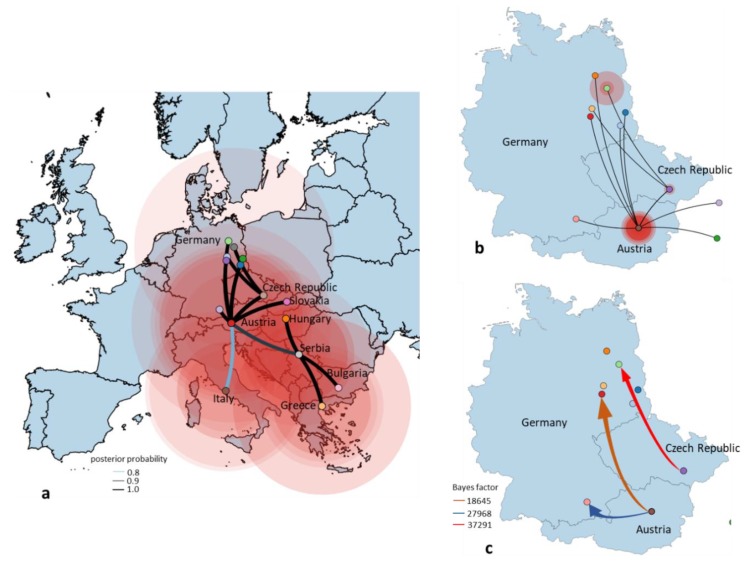
Spatial dynamics of the European clade of WNV lineage 2 including the origin of the German WNV reconstructed from the Bayesian maximum clade credibility (MCC) tree, a flexible demographic prior with location states and a Bayesian Stochastic Search Variable Selection (BSSVS); (**a**) the directed lines between locations connect the sources and target countries. Circles represent discrete geographical locations of viral strains and represent branches in the MCC tree along with where the relevant location transition occurs. All introductions for Germany are shown. Circle diameters of locations are proportional to square root of the number of MCC branches maintaining a particular location state at each time-point. Discrete locations are geographic coordinates for each European country; (**b**) the directed lines between the source of viral strains (Czech Republic and Austria) and target locations in Germany. Location circle diameters are proportional to square root of the number of MCC branches maintaining a particular location state at each time-point; (**c**) migration pattern of WNV between Czech Republic–Germany and Austria–Germany based on Bayes factor (BF) test for significant non-zero rates. Viral migration patterns are indicated between the different regions of Germany and neighboring countries and are proportional to the strength of the transmission rate. The color of the connections indicates the origin and the direction of migration and are proportional with the strength of connections. Only well supported paths between locations are shown.

Based on the albeit only limited Central European strain data, the tMRCA of the EGC group indicates a very recent emergence which was most likely introduced into Germany as a single introduction event. The progenitor of this Eastern European clade dates back to 2011, most probably circulating in Czech Republic (95% HPD 2010–2012; posterior probability, pp = 0.88) (Figure 2a). The EGC shares a common ancestor with basal WNV from Germany providing strong support for in situ evolution of WNV in Germany (Figure 3 and Figure 4). Except for the members of the EGC, all other WNV strains found most recently in Germany seem to be descendants of ancestors from Austria (95% HPD for 2000 to 2015; pp = 0.83−0.97). The spatial diffusion pattern of WNV within Germany and between Germany and neighboring countries has been reconstructed using a Bayes Factor (BF) test under Bayesian Stochastic Search Variable Selection analysis (BSSVS). The strongest epidemiological links based on the BF estimates have been detected between Austria–Germany and Czech Republic–Germany, while the links within Germany have been detected between Halle–Berlin, Berlin–Halle, Berlin–Hamburg, Berlin–Dresden and Halle with neighboring localities (Figure 5 and Figure 6). Similar star-like relationships of the WNV as for EGC have been also observed for Italian and Greek strains within both, SEEC and CEC (Figure 2a and Figure 3). These results further provide indication for the in situ evolution of the European lineages.

### 3.5. Population Dynamics, Protein Changes and Analysis of Selection Pressure

**Figure 7:**
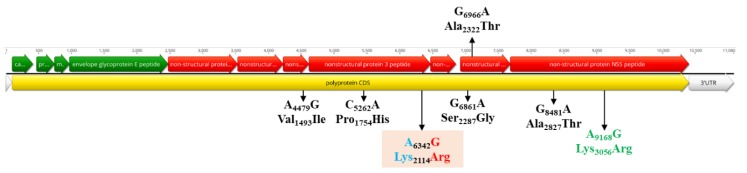
Schematic representation of the WNV genome and the positions of amino acid mutations. The position of the unique amino acid mutation of the Eastern German clade (colored in red/blue) in the NS3 gene is highlighted. The specific non-synonymous amino acid mutations for the CEC are shown in black, while the mutation in the NS5 specific for the subclade including the Eastern German group, one Austrian, one Czech and two German strains is presented in green.

The mean rate of evolution estimated for the polyptrotein of the EGC was 1.26 × 10^−4^ (95% HPD, 1.15 × 10^−5^–2.84 × 10^−4^) subs site^−1^ year^−1^ two times lower than for the European data set, 2.51 × 10^−4^ (95% HPD, 2.13 × 10^−4^–2.88 × 10^−4^) subs site^−1^ year^−1^ (Figure 2c). EGC population dynamics showed a slightly increased growth phase from the beginning of emergence when the virus effective population size (Ne) remained constant until 2015. From that year, a constant increasing tendency for the Ne values was observed, which is in line with the strong population expansion started in 2015–2016 (Figure 2a,b). The monophyletic LysArg mutation located in the C terminus of the NS3 gene appeared only in Eastern German clade strains, while the paraphyletic Lys_3056_Arg mutation from the NS5 gene was found to be common for EGC strains and some WNV from Austria (MF984341), Czech Republic (KM203862) and Germany (LR743437 and LR743434). There are several non-synonymous mutations in the nonstructural genes, which exhibit geographical structures specific of the members of the CEC (Figure 7). The overall d_N_/d_S_ ratios in the polyprotein of EGC, CEC and SEEC were 0.118, 0.136 and 0.154, respectively, indicating that most sites are subject to strong purifying or negative selection. There was no evidence for positive or episodic diversifying selection in the WNV strains from Germany.

## 4. Discussion

Globalization and climate change enhance or can lead the migration of exotic pathogens and their hosts to new environments promoting the contacts with naïve and vulnerable hosts. Thus, understanding the local ecological factors and evolutionary processes which navigate the emergence, establishment and spread of newly introduced viral diseases is critical for developing and implementing surveillance strategies for disease control. The present study aimed to elucidate the possible origins, spatiotemporal spread pattern tendencies and eco-epidemiological factors that facilitate WNV becoming an established pathogen in Germany causing neuroinvasive disease in multiple vertebrate species, including humans.

One year after the first observed autochthonous WNV transmission to birds and horses in Germany [9,13], an epizootic emergence of WNV was again observed in 2019. The number of infected birds and horses was considerably higher (76 birds and 36 horses) compared to 2018 (12 birds and two horses). In contrast to the USA, WNV-associated mass mortality in birds had not been observed in Europe before [33]. Previous hypotheses for this difference have been refuted by several research studies, e.g., demonstrating that European birds are susceptible to WNV infections and *Culex* mosquitoes in Europe are competent to transmit WNV. An alternative explanation might by that the bird mortality is so low that it is not detected with current European surveillance programs. A comprehensive USUV/WNV monitoring system is in place, but we also only see the tip of the iceberg of WNV infected birds in Germany. In addition, a huge number of positive specimens were obtained from captive animals (e.g., birds kept in zoos), which have a higher probability of detection compared to wild animals. Furthermore, from these birds, a considerable proportion were birds of prey, which must be considered to have a higher susceptibility to WNV infection [34]. This in combination with a widespread enzootic circulation of WNV and large number of equine cases—36 in 2019 in contrast to 2 in 2018—indicates an increased risk of WNV spillover into the human population.

This is reflected in the detection of five laboratory confirmed, mosquito-borne, autochthonous human WNV cases in 2019. It has to be kept in mind that less than 25% of infected humans develop noticeable symptoms [3]. Even fewer patients (<1%) have a risk of developing WNND [4]. The number of observed WNND cases (three of the five confirmed human WNV) gives rise to the speculation that hundreds of undetected human WNV infections in Germany occurred during the epidemic in 2019.

WNV transmission and spread is significantly influenced by climatic conditions, e.g., shaping phenology and abundance of the vector. Temperature is one of the most important factors directly affecting the EIP in different mosquito vector species [14]. High daily average temperatures (> 20 °C) over several days are required to allow for WNV transmission, which is correlated to the main distribution of WNV in south-eastern Europe. This also matches the spatial pattern of WNV in Germany. The summers in 2018 and 2019 were both characterized by extraordinarily high temperatures allowing low EIP values. The area in central-eastern Germany as the main focus of WNV circulation in both years was characterized by shorter EIP compared to previous years and most other areas in Germany [13]. Furthermore, these areas in Germany are directly neighboring countries reporting several years of WNV circulation (e.g., Czech Republic), leading to a high risk of short distance introductions e.g., by infected birds. The analysis also indicated that the areas along the Upper Rhine Valley in south-west Germany had a high suitability for WNV circulation, but no WNV activity was observed in all previous surveillance programs [8,9,10,11]. Most likely, no WNV introduction and circulation occurred yet, which underpins the thesis of the entries over short distances. Future studies are needed to understand if the virus did not yet spread to this area or if there are other factors reducing the risk of virus circulation (e.g., distribution of suitable vector or host species). The phylogenetic analyses indicated that Germany experienced at least six distinct WNV introduction events, with Austria and Czech Republic as possible origin for the progenitors of the German WNV epizootic strain variants. The majority of these strains clustered together into a distinct subclade (EGC). 

The ongoing circulation and dominance of the EGC detected in 2019 indicates successful overwintering of WNV in Germany, e.g., through WNV persistence in hibernating mosquitoes throughout the winter season [35]. The virus variants of the EGC at multiple sites detected in the epidemic in 2019 are descendants of a common ancestor in the wider central European environment which dates back to the time span 2010–2012. Where and when the subsequent virus evolution to the current variants took place and how descendants were eventually transferred to Germany remains elusive. However, such a translocation and subsequent virus amplification may have been fostered by the extremely favorable climatic conditions for mosquitos in Germany in spring/summer 2018, and the short distance transmission with infected birds from neighboring countries.

There has been a comprehensive USUV/WNV monitoring system in place in Germany for over a decade which involves ornithologists, zoological gardens and bird clinics supplying thousands of zoo and wild bird samples for WNV antibody and genome analysis. Moreover, there has been an exhaustive mosquito surveillance in place in Germany since 2009. By both surveillance approaches a variety of viruses were found, such as Sindbis virus, Batai virus and USUV, but not WNV [9,10,11,13,36,37]. At the same time, different long-distance, partial and short-distance migratory birds showed neutralizing antibodies against WNV before 2018 [9,11]. Although any such monitoring scheme has its predictive limitations due to sampling size constraints, all the negative WNV monitoring results from birds, horses and mosquitos before 2018 and the proximity to a larger region with active WNV circulation supports a recent introduction of multiple WNV descendants e.g., from Czech Republic to Germany. However, sequencing a larger number of more current WNV strains from e.g., Austria or the Czech Republic would help to answer the circumstances of when and what in regard to the development of the East German Clade variants. Overall, the number of recent whole genome sequences is limited and should be markedly increased using NGS-based approaches. 

Most of the singleton WNV variants in Germany do not contain the monophyletic Lys2114Arg mutation located in the C terminus of the NS3 gene, even if these strains circulate in the central-eastern part of the country with very high WNV activity and rapid expansion of the EGC. Although these singletons have circulated and evolved under the same ecological conditions as members of EGC, it seems that these variants were not able to perpetuate and establish a stable enzootic cycle leading to a similar epizootic/epidemic scenario as for the EGC group. In case of the EGC, the adaptation to naïve vector and host populations leads to the emergence of local virus variants. The most likely scenario for EGC might be enzootic maintenance similar to that observed for WNV in the United States [38,39]. This hypothesis is supported by the observation that EGC form a star-like structure (population expansion after a single viral introduction) in which the variant viral strains accumulate changes during the rapid adaptation to the local ecological conditions (adaptation of the virus to the host populations and its enzootic maintenance), as observed for Usutu virus [40]. 

We found evidence that the phylogenetic structure of EGC and virus genetic population growth is shaped by the geographic location and average extrinsic incubation period, which likely facilitated rapid short-distance virus dispersal in 2018/2019. This demonstrates that local ecological factors (e.g., average temperature profile during the vector season) could predict the local and regional dispersal patterns of WNV in our data sets. 

The purifying and negative selections observed for WNV in Germany were expected given the transmission and infection modes of arboviruses, leading to the accumulation of synonymous mutations [41]. Mutation observed at amino acid position Lys2114Arg has been found to be involved in the formation of EGC, while Val1493Ile (NS2b), Pro1754His (NS3), Ser2287Gly and Ala2322Thr (NS4b), Ala2827Thr and Lys3056Arg (NS5) are specific for the CEC (convergent evolution). Similar patterns of parallel or convergent evolution have been observed for WNV. This suggests that a limited number of residue changes are permitted due to functional constraints [42]. Viral adaptation in vector and vertebrate hosts by local overwintering or reintroduction of the virus and local ecological conditions (e.g., high average EIP) could be considered key determinants in the spatial dispersal and establishment of WNV. It is interesting to note that the Lys2114Arg mutation is specific for the newly described EGC. The impact of this mutation is unclear; a similar change in the WNV NS3 helicase (Thr1754Pro) generated a highly virulent phenotype to American crows [43]. In vitro and in vivo experiments with strains from the EGC might show the role of fitness and pathogenicity in the future. 

## 5. Conclusions

This study provides a first comprehensive summary and phylogeographic analysis on the WNV epidemic in Germany in 2018 and 2019 and highlights the risk of human WNV infections causing considerable risks for transfusion and organ transplantation safety. Therefore, intensive surveillance of mosquitoes, birds, horses and humans should remain a public health priority, e.g., to monitor the occurrence and subsequent spread of WNV or to develop targeted control mechanisms. Our study also highlights the need for international cooperation in the area of WNV surveillance and monitoring, especially across national borders and as a “one-health” approach for an improved risk analysis. This should also include the generation of higher numbers of whole-genome sequences, allowing for a more precise molecular epidemiology and strain characterization.

## Figures and Tables

**Table 1:** West Nile virus (WNV)-positive birds, horses and mosquito-borne, autochthonous humans for the federal states of Germany in 2018/2019. Numbers in brackets indicate the number of samples with WNV sequences acquired in this study.

Federal State	Birds (2018)	Horses (2018)	Birds (2019)	Horses (2019)	Humans (2019)	Sum
Bavaria (BY)	2 (2)	0	0	0	0	**2 (2)**
Berlin (BE)	3 (1)	0	33 (6)	0	1 (1)	**37 (8)**
Brandenburg (BB)	0	1	6 (3)	7	0	**14 (3)**
Hamburg (HH)	0	0	1 (1)	0	0	**1 (1)**
Mecklenburg-Western Pomerania (MV)	1	0	0	0	0	**1**
Saxony (SN)	1 (1)	0	21 (8)	9 (1)	3	**34 (10)**
Saxony-Anhalt (ST)	5 (2)	1	15 (10)	19	1 (1)	**41 (13)**
Thuringia (TH)	0	0	0	1	0	**1**
**Sum**	**12 (6)**	**2**	**76 (28)**	**36 (1)**	**5 (2)**	**131 (37)**

In addition to the 37 WNV sequences, two more genome sequences were obtained from WNV-positive mosquito pools collected in Berlin.

**Table 2:** Detection of WNV infections in different bird species in 2018 and 2019.

Bird Species	Scientific Name	Housing	Number of WNV-Infected Birds	Affected Federal States *
Eurasian Blackbird	*Turdus merula*	wild	3	ST, MV
Andean Flamingo	*Phoenicoparrus andinus*	captive	1	BE
Great Grey Owl	*Strix nebulosa*	captive	6	SN, ST, BY
Unspecified buzzard	*Buteo* sp.	wild	1	ST
Blue Tit	*Parus caeruleus*	wild	3	SN, ST
Chilean Flamingo	*Phoenicopterus chilensis*	captive	6	BE, SN
Eurasian Jay	*Garrulus glandarius*	wild	1	BB
Coconut Lorikeet	*Trichoglossus haematodus*	captive	1	ST
Scarlet-chested Parrot	*Neophema splendida*	captive	1	SN
Eurasian Golden Plover	*Pluvialis apricaria*	wild	1	SN
Northern Goshawk	*Accipiter gentilis*	wild/captive	19	BB, BE, SN, ST
House Sparrow	*Passer domesticus*	wild	4	SN, ST
Dunnock	*Prunella modularis*	wild	1	HH
Humboldt-Penguin	*Spheniscus humboldti*	captive	1	BB
Inka-Tern	*Larosterna inca*	captive	1	BE
Black-tailed Gull	*Larus crassirostris*	captive	8	BE
Kagu	*Rhynochetos jubatus*	captive	1	BE
Domestic Canary	*Serinus canaria forma domestica*	captive	2	SN
Great Tit	*Parus major*	wild	3	SN
American Flamingo	*Phoenicopterus ruber*	captive	3	BE
Hooded Crow	*Corvus corone cornix*	wild	1	BE
Unspecified pelican	*Pelecanus* sp.	captive	1	ST
Javan Pond Heron	*Ardeola speciosa*	captive	1	BE
Common Wood Pigeon	*Columba palumbus*	wild	1	BE
Snowy Owl	*Bubo scandiacus*	captive	8	BE, ST
Chinese Merganser	*Mergus squamatus*	captive	1	BE
Swift Parrot	*Lathamus discolor*	captive	1	SN
Little Owl	*Athene noctua*	wild	2	BB
European Goldfinch	*Carduelis carduelis*	captive	1	SN
Eurasian Eagle-Owl	*Bubo bubo*	wild	1	SN
Tawny Owl	*Strix aluco*	wild	1	ST
White Eared Pheasant	*Crossoptilon crossoptilon*	captive	2	BE

* abbreviations as in Table 1.

## Supplementary Materials

The following are available online at https://www.mdpi.com/1999-4915/12/4/448/s1, Table S1: Epidemiological data of West Nile virus with full genome sequences (except for 1 human sample), their corresponding accession numbers and sequencing protocol performed.
