# Supplementary material for: West Nile Virus Epidemic in Germany Triggered by Epizootic Emergence, 2019
Source: Viruses. 2020 Apr 15;12(4):448. doi: 10.3390/v12040448 (PMC7232143; doi:10.3390/v12040448)
Supplement: Supplementary file 1 [file viruses-12-00448-s001.pdf]

Table S1. Epidemiological data of West Nile virus with full genome sequences (except for 1 human sample), their corresponding accession numbers and sequencing protocol performed.

| Accession Number | Virus identifier | Library number | Host common name | Host scientific name | City | Region | Sequencing Protocol |
|---|---|---|---|---|---|---|---|
| LR743437 | ED-I-90_18 | lib02896 | Great Grey Owl | *Strix nebulosa* | Poing | Bavaria | Direct NGS |
| LR743434 | ED-I-114_18 | lib02898 | Great Grey Owl | *Strix nebulosa* | Poing | Bavaria | Direct NGS |
| LR743433 | ED-I-82_18 | lib02914 | Goshawk | *Accipiter gentilis* | Klein Weissandt | Saxony-Anhalt | Direct NGS |
| LR743429 | ED-I-142_18 | lib02916 | Tawny Owl | *Strix aluco* | Bad Lauchstaedt | Saxony-Anhalt | Direct NGS |
| LR743436 | ED-I-89_18 | lib02959 | Goshawk | *Accipiter gentilis* | Bad Düben | Saxony | Direct NGS |
| LR743443 | ED-I-107-18 | lib03041 | Snowy Owl | *Bubo scandiacus* | Berlin Friedrichsfelde | Berlin | Direct NGS |
| LR743425 | ED-I-62_19 | lib03378 | Snowy Owl | *Bubo scandiacus* | Lutherstadt Wittenberg | Saxony Anhalt | Direct NGS |
| LR743444 | ED-I-83_19 | lib03379 | Great Grey Owl | *Strix nebulosa* | Lutherstadt Wittenberg | Saxony Anhalt | Direct NGS |
| LR743424 | ED-I-85_19 | lib03380 | Snowy Owl | *Bubo scandiacus* | Berlin Friedrichsfelde | Berlin | Direct NGS |
| LR743442 | ED-I-87_19 | lib03381 | Blue Tit | *Parus caeruleus* | Halle (Saale) | Saxony-Anhalt | Direct NGS |
| LR743428 | ED-I-118_19 | lib03382 | Snowy Owl | *Bubo scandiacus* | Berlin Friedrichsfelde | Berlin | Direct NGS |
| LR743426 | ED-I-157_19 | lib03415 | Snowy Owl | *Bubo scandiacus* | Berlin | Berlin | Direct NGS |
| LR743432 | ED-I-158_19 | lib03416 | Andean Flamingo | *Phoenicoparrus andinus* | Berlin | Berlin | Direct NGS |
| LR743427 | ED-I-148_19 | lib03417 | Goshawk | *Accipiter gentilis* | Berlin | Berlin | Direct NGS |
| LR743423 | ED-I-156_19 | lib03418 | Goshawk | *Accipiter gentilis* | Berlin Friedrichsfelde | Berlin | Direct NGS |
| LR743435 | ED-I-153_19 | lib03419 | Goshawk | *Accipiter gentilis* | Brieselang | Brandenburg | Direct NGS |
| LR743422 | ED-I-155_19 | lib03420 | Goshawk | *Accipiter gentilis* | Neuruppin | Brandenburg | Direct NGS |
| LR743430 | ED-I-89_19 | lib03421 | Blue Tit | *Parus caeruleus* | Radebeul | Saxony | Direct NGS |
| LR743421 | ED-I-139_19 | lib03422 | Great Tit | *Parus major* | Dresden | Saxony | Direct NGS |
| LR743431 | ED-I-177_19 | lib03423 | Eurasian Golden Plover | *Pluvialis apricaria* | Kamenz/Biehla | Saxony | Direct NGS |
| LR743449 | ED-I-163_19 | lib03424 | Goshawk | *Accipiter gentilis* | Sandersdorf | Saxony-Anhalt | Direct NGS |
| LR743458 | ED-I-164_19 | lib03425 | Snowy Owl | *Bubo scandiacus* | Magdeburg | Saxony-Anhalt | Direct NGS |
| LR743451 | ED-I-165_19 | lib03426 | Snowy Owl | *Bubo scandiacus* | Magdeburg | Saxony-Anhalt | Direct NGS |
| LR743446 | ED-I-109_19 | lib03427 | Coconut Lorikeet | *Trichoglossus haematodus* | Halle (Saale) | Saxony-Anhalt | Direct NGS |
| LR743456 | ED-I-134_19 | lib03428 | House Sparrow | *Passer domesticus* | Rackwitz | Saxony | Direct NGS |
| LR743445 | ED-I-172_19 | lib03430 | Great Grey Owl | *Strix nebulosa* | Chemnitz | Saxony | Direct NGS |
| LR743452 | ED-I-173_19 | lib03431 | Great Grey Owl | *Strix nebulosa* | Chemnitz | Saxony | Direct NGS |
| LR743450 | ED-I-202_19 | lib03432 | Chilean Flamingo | *Phoenicopterus chilensis* | Leipzig | Saxony | Direct NGS |
| LR743454 | ED-I-205_19 | lib03433 | Great Tit | *Parus major* | Bad Düben | Saxony | Direct NGS |
| LR743448 | ED-I-201_19 | lib03449 | Humboldt-Penguin | *Spheniscus humboldti* | Cottbus | Brandenburg | Direct NGS |
| LR743457 | ED-I-208_19 | lib03450 | Goshawk | *Accipiter gentilis* | Merseburg | Saxony-Anhalt | Direct NGS |

| | | | | | | | |
|---|---|---|---|---|---|---|---|
| LR743453 | ED-I-94_19 | lib03451 | Horse | *Equus ferus caballus* | Krostitz | Saxony | Direct NGS with MyBaits treatment |
| LR743455 | C6_T167_20 | lib03481 | Mosquito | *Culex pipiens* | Berlin Friedrichsfelde | Berlin | Direct NGS |
| LR743447 | C6_T167_57 | lib03482 | Mosquito | *Culex pipiens* | Berlin Friedrichsfelde | Berlin | Direct NGS |
| MN794935 | BNI-10/19 | BNI42493 | Human | *Homo sapiens* | Leipzig | Saxony | Direct NGS |
| MN794936 | BNI-Berlin748 | BNI45435 | Human | *Homo sapiens* | Berlin | Berlin | Direct NGS |
| MN794937 | BNI-2635 | BNI41487 | Blackbird | *Turdus merula* | Schönebeck | Saxony-Anhalt | Direct NGS |
| MN794938 | BNI-2615 | BNI41323 | House sparrow | *Passer domesticus* | Jeßwitz | Saxony-Anhalt | Direct NGS |
| MN794939 | BNI-2432 | BNI40119 | Dunnock | *Prunella modularis* | Hamburg | Hamburg | Direct NGS |
